# CircRNA has_circ_0017109 promotes lung tumor progression via activation of Wnt/β-catenin signaling due to modulating miR-671-5p/FZD4 axis

**DOI:** 10.1186/s12890-022-02209-2

**Published:** 2022-11-24

**Authors:** Bo Yang, Bin Zhang, Qi Qi, Changli Wang

**Affiliations:** 1 Department of Lung Cancer, Tianjin Medical University Cancer Institute and Hospital, Huan-hu-xi Road, Ti-Yuan-Bei, He XI Disrict, Tianjin, 30060 P.R. China; 2 National Clinical Research Center for Cancer, Key Laboratory of Cancer Prevention and Therapy, Huan-hu-xi Road, Ti-Yuan-Bei, He XI Disrict, Tianjin, 30060 P.R. China; 3 Tianjin’s Clinical Research Center for Cancer, Tianjin Lung Cancer Center, Huan-hu-xi Road, Ti-Yuan-Bei, He XI Disrict, Tianjin, 30060 P.R. China

**Keywords:** circRNA, NSCLC, Lung cancer, miRNA, FZD4, Wnt/β-catenin

## Abstract

**Introduction:**

Accumulating evidence highlights the critical roles of circular RNAs (circRNAs) in the malignant progression of cancers. In this study, we investigated the expression pattern of a newly identified circRNA (hsa_circ_0017109) in non–small cell lung cancer (NSCLC), and examined its downstream molecular targets.

**Methods:**

Quantitative real-time PCR (qRT-PCR) and Western blotting (WB) were conducted to quantify gene and protein expression. In vitro functional assays such as colony formation assay, cell counting kit-8 (CCK-8) and flow cytometry were used to study cell proliferation and apoptosis. RNA pull-down assay, luciferase reporter assay and RNA immunoprecipitation were performed to validate molecular interaction. Mouse xenograft model of NSCLC cells was used to assess the role of circ_0017109 in tumorigenesis.

**Results:**

Circ_0017109 was upregulated in NSCLC tumor samples and cells. Silencing circ_0017109 impaired cell proliferation and promoted apoptosis in NSCLC cells, and circ_0017109 knockdown suppressed in vivo tumorigenesis of NSCLC cells in mouse xenograft model. MiR-671-5p was identified as a target of circ_0017109, and circ_0017109 negatively impacted on miR-671-5p expression. MiR-671-5p downregulated FZD4 and dampened the activity of Wnt/β-catenin signaling pathway. Circ_0017109 modulated FZD4 expression by suppressing miR-671-5p activity.

**Conclusions:**

Elevated circ_0017109 expression promotes tumor progression of NSCLC by modulating miR-671-5p/FZD4/β-catenin axis.

**Supplementary Information:**

The online version contains supplementary material available at 10.1186/s12890-022-02209-2.

## Introduction

Non–small cell lung cancer (NSCLC) is a dominant subtype of lung cancers, accounting for approximately 85% of total diagnoses in lung cancer [1]. Unfortunately, the diagnosis of NSCLC is often made in the late stage due to insidious and asymptomatic nature of the disease [1]. Due to the poor prognosis of patients with advanced NSCLC, the 5 years’s survival rate of NSCLC patients is dismay despite the advances in treatment strategies [2]. To achieve satisfactory treatment outcome, early diagnosis is critical for optimal surgery and the choice of effective adjuvant therapy. Identification of diagnostic and prognostic biomarkers for NSCLC is imperative for the early diagnosis and the selection of effective treatment scheme. The wide application of next-generation sequencing technologies enables the characterization of gene regulatory networks in cancer. Circular ribonucleic acid (circRNAs) emerges as a novel family of non-coding RNAs which are recognized as potential diagnostic biomarkers for certain cancers [3].

CircRNAs are previously considered as aberrant by-products of messenger RNA splicing; however, recent studies clarified that they play pivotal regulatory roles in multiple cellular processes [3]. These functional molecules exist as closed-loop structure resulted from the back-splicing, and hence show high stability. CircRNAs are broadly expressed in mammalian cells with tissue-specific and cell-specific pattern. Recent efforts unveiled different mechanisms of action of circRNAs: they may act as microRNA (miRNA) sponges or decoys; they may bind to certain target protein to modulate their activity; or occasionally they are translated into small peptides [4]. The best-characterized biological activity of circRNAs is the molecular sponge of miRNA. Each circRNA can harbor multiple miRNA binding sites and the physical absorption of miRNAs affects the activity and stability of miRNAs. Dysregulation of circRNAs has been implicated in the development of gastric cancer (GC) [5, 6], colorectal cancer (CRC) [7, 8], ovarian [9], bladder [10], and breast cancer (BC) [11]. It has been also noticed that the same circRNA may display different mode of actions in different types of cancers.

Has_circ_0017109 is one of the most recently identified circRNAs, which may play a critical function in lung cancer occurrence and development. Initially, our analyses on the microarray dataset profiling non-coding RNAs in NSCLC tissues and adjacent normal tissues (GSE101586 and GSE112214) revealed that, circ_0017109 expression was significantly higher in NSCLC tumors samples in comparison to the matched normal samples. The functional role of circ_0017109, however, has not yet been studied in NSCLC. Previous studies identified miR-671-5p as the miRNA target of circ_0017109, and miR-671-5p expression was suppressed within several malignant tumors, like osteosarcoma [12], BC [13, 14], and glioblastoma (GBM) [15]. Interestingly, frizzled-4 (FZD4, the upstream regulator of Wnt/β-catenin) was downregulated in the above process, along with the suppression of Wnt/β-catenin signaling [16, 17]. In this study, we first investigated the functional role of circ_0017109 in regulating the malignant phenotype and tumorigenesis of NSCLC cells. We further validated the functional interaction of circ_0017109 and miR-671-5p, and demonstrated that miR-671-5p/FZD4 axis mediated the functional role of circ_0017109.

## Results

### Circ_0017109 is upregulated in NSCLC and associated with the prognosis of NSCLC patient

**Fig. 1:**
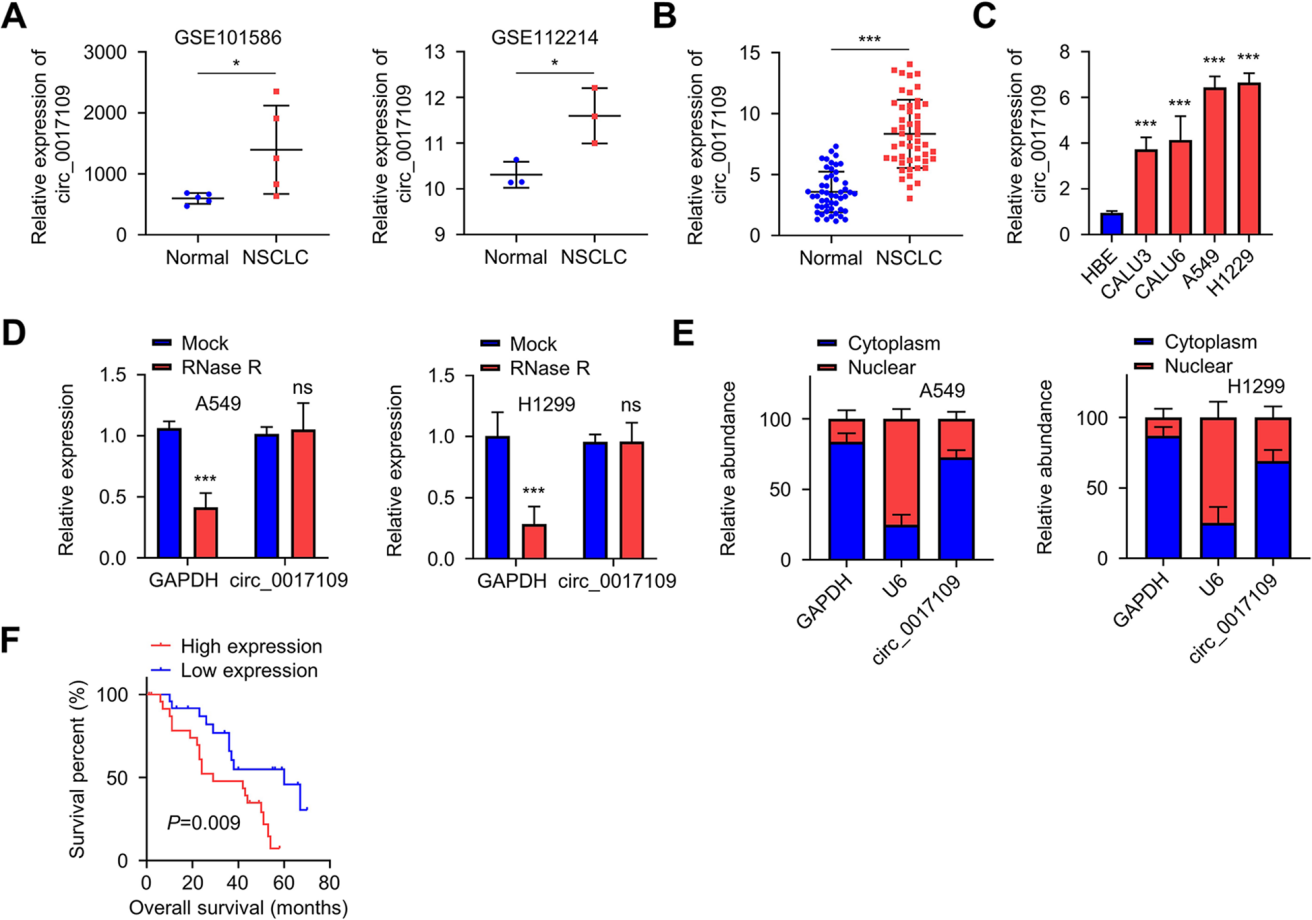
Circ_0017109 is upregulated in NSCLC and associated with poor prognosis in NSCLC patients. **A** Expression of circ_0017109 in normal lung tissues and NSCLC tumor samples of GSE101586 and GSE112214 (GEO). **B** Circ_0017109 levels within 50 pairs of NSCLC samples and matched non-carcinoma samples. **C** Circ_0017109 levels within NSCLC cells, (A549, CALU3, H1229 and CALU6) and human normal bronchial epithelial cells (HBE). **D** GAPDH and circ_0017109 levels in the RNA samples from H1229 and A549 cell lines with or without RNase R treatment. **E** RNA levels of circ_0017109, U6 (nuclear control), and GAPDH (cytoplasmic control) in the cytoplasmic and nuclear fraction of H1229 and A549 cells. **F** The association of NSCLC patient overall survival and circ_0017109 expression level. *, *P* < 0.05, and ***, *P* < 0.001

Through analyzing GSE101586 and GSE112214 microarray data of non-coding RNA profiling in NSCLC tumor and normal tissues, we found that circ_0017109 showed higher expression in NSCLC samples when compared to normal tissue (Fig. 1A). We then collected 50 pairs of NSCLC samples and the matched non-carcinoma samples, and qRT-PCR assay confirmed the upregulation of circ_0017109 in NSCLC (Fig. 1B). Accordingly, circ_0017109 level was elevated in NSCLC cells (A549, CALU3, H1229, CALU6) when compared to human bronchial epithelial cells (HBE, Fig. 1C). To confirm the circularity of circ_0017109, RNA samples extracted from A549 and H1229 cells were treated with RNase R. Compared to the linear mRNA of glyceraldehyde 3-phosphate dehydrogenase (GAPDH) which was degraded by RNase R, circ_0017109 was highly resistant to RNase R digestion (Fig. 1D). According to the quantification of the nuclear and cytoplasmic fractions, circ_0017109 showed a predominant localization in the cytoplasmic fraction in A549 and H1229 cells (Fig. 1E).

Additionally, we analyzed the clinicopathological features of 50 patients with NSCLC and examined the association with circ_0017109 expression level. The median expression value of circ_0017109 was used as the cut-off to assign the patients into low- and high-expression groups (Table 1). We found a significant association between high expression of circ_0017109 and tumor size, lymph node metastasis (LNM) and TNM stages (*p* < 0.05). Next, Kaplan-Meier (K-M) plot was adopted to analyze the prognostic value of circ_0017109 level in patients with NSCLC. High level of circ_0017109 expression was associated with an worse overall survival among NSCLC cases (*p* < 0.01; Fig. 1F). Taken together, circ_0017109 was upregulated within NSCLC, which showed positive correlations with malignant features of NSCLC. These results highlight the potential applicability of circ_0017109 as a prognostic biomarker in NSCLC.

**Table 1:** Correlation between clinicopathological characteristics of NSCLC patients and circ_0017109 expression level

Clinicopathological features		No.	Low circ_0017109 expression (*n* = 25)	High circ_0017109 expression (*n* = 25)	*P-*value
Age	≥65	26	12	14	0.5713
	<65	24	13	11	
Gender	Male	22	10	12	0.5688
	Female	28	15	13	
Tumor size	≥3 cm	23	8	15	**0.047**
	<3 cm	27	17	10	
Differentiation	Well-Moderate	32	19	13	0.0771
	Poor	18	6	12	
Lymph node metastasis	Positive	15	4	11	**0.0308**
	Negative	35	21	14	
TNM stage	I + II	28	18	10	**0.0227**
	III + IV	22	7	15	

### Circ_0017109 is essential to support the proliferation and survival of NSCLC cells

**Fig. 2:**
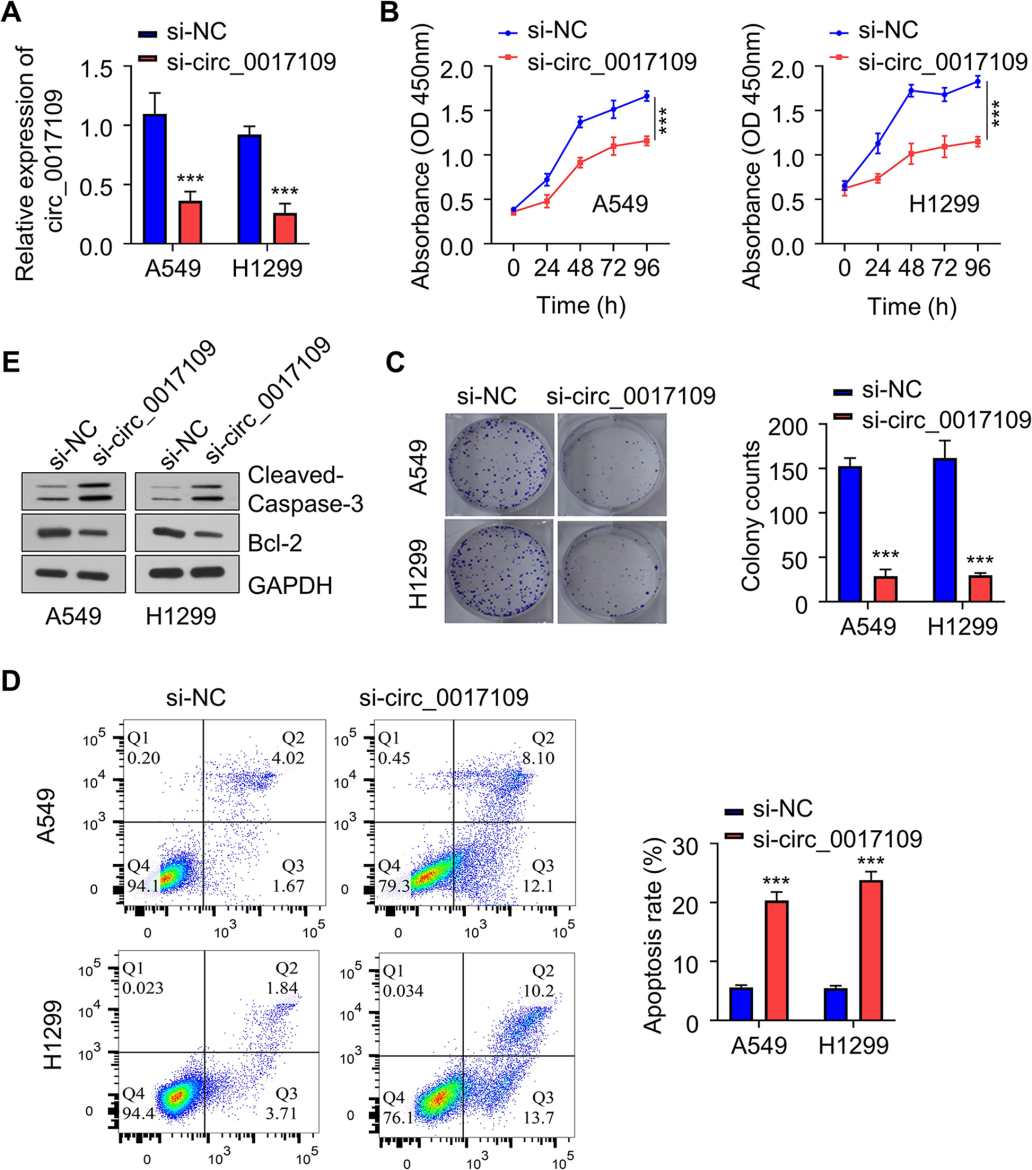
Circ_0017109 promotes cell growth and inhibits apoptosis of NSCLC cells. **A** Circ_0017109 expression within H1299 and A549 cells after the transfection of si-circ_0017109 or si-NC. **B** CCK-8 assay in H1299 and A549 cells after the transfection of si-circ_0017109 or si-NC. **C** Colony formation assay in H1299 and A549 cells after the transfection of si-circ_0017109 or si-NC. **D** Apoptosis detection in H1299 and A549 cells after the transfection of si-circ_0017109 or si-NC. **E** Protein levels of cleaved-caspase-3 and Bcl-2 in H1299 and A549 cells after the transfection of si-circ_0017109 or si-NC. **, *P* < 0.01, and ***, *P* < 0.001

To study the function of circ_0017109 in NSCLC cells, we applied siRNA targeting circ_0017109 in A549 and H1299 cells. After transfection, circ_0017109 level was significantly reduced in both cell lines (Fig. 2A). CCK-8 assay showed that silencing circ_0017109 suppressed cell growth in comparison to the control group (Fig. 2B). Consistently, colony forming experiment suggested the transfection with si_circ_0017109 significantly reduced colony forming capacity in both cells (Fig. 2C). In circ_0017109-silenced cells, we observed an increased proportion of apoptotic cells in relative to cells transfected with si-NC (Fig. 2D). We also evaluated the apoptosis-related proteins such as B-cell lymphoma-2 (Bcl-2) and cleaved-caspase-3. Circ-0017109 knockdown promoted the cleavage of caspase-3 (pro-apoptotic) while Bcl-2 (anti-apoptotic) level was downregulated (Fig. 2E). To strengthen our finding, we also applied a second siRNA to knock down circ_0017109 in A549 and H1299 cells. The results further validated that downregulating circ_0017109 undermines cell proliferation and induced apoptosis in NSCLC cells (Fig. S1A-C). Altogether, these findings suggest an indispensable role of circ_0017109 in sustaining cell proliferation and survival in NSCLC cells.

### miR-671-5p is directly regulated by circ_0017109

To find the potential targets of circ_0017109, we searched the CircInteractome database (https://circinteractome.nia.nih.gov/) and identified multiple potential miRNA targets of circ_0017109 (Fig. S2A). To narrow down the targets of circ_0017109, we performed qRT-PCR analysis of these miRNAs between NSCLC cells and human bronchial epithelial HBE cells. The results revealed that only miR-671-5p showed downregulation in A549 and H1299 cells, when compared to HBE cells (Fig. S2B). Since circ_0017109 was upregulated in A549 and H1299 cells, these data indicate a potential sponging effect of circ_0017109 on miR-671-5p. We therefore selected miR-671-5p as the candidate target of circ_0017109 for the following study.

**Fig. 3:**
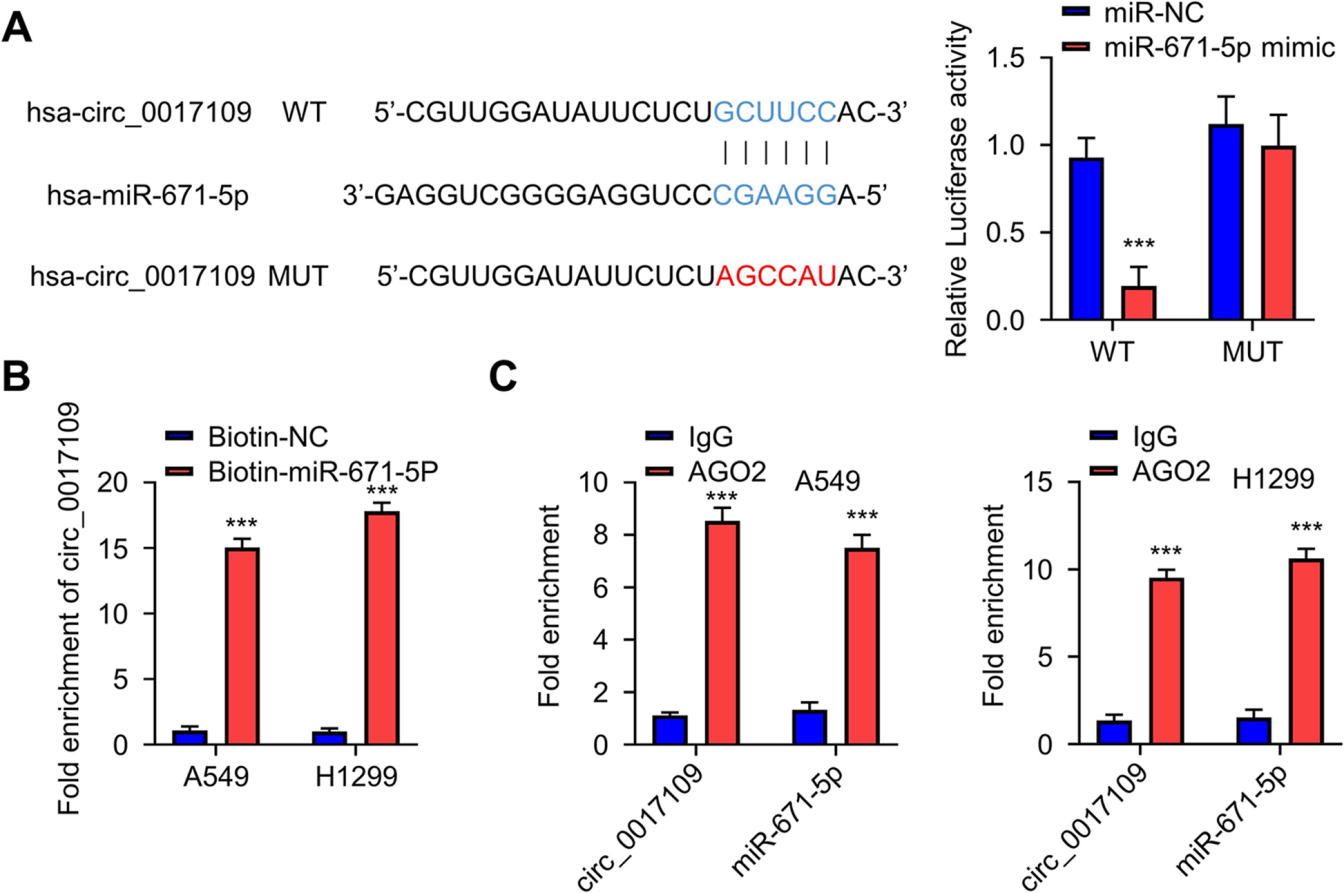
MiR-671-5p is regulated by circ_0017109. **A** Predicted miR-671-5p target sequences in circ_0017109 sequence based on CircInteractome database. Luciferase activity of wide-type or mutated circ_0017109 reporter was determined in H293K cells in the presence of miR-NC or miR-671-5p mimic. **B** RNA-pull down assay of circ_0017109 enrichment using biotinylated miR-671-5p or biotin-NC. **C** RNA-immunoprecipitation assay using anti-AGO2 antibody or nonspecific rabbit IgG. ***, *P* < 0.001

We cloned the wild type binding sites in circ_0017109 (WT) or mutated binding sequences (MUT) into a luciferase reporter to study their potential functional interaction (Fig. 3A). In H293T cells, the transfection of miR-671-5p mimic suppressed luciferase activity of WT reporter, while in MUT reporter miR-671-5p mimic showed no suppression (Fig. 3A). The data suggest that circ_0017109 and miR-671-5p interact through the predicted binding sequence. We further conducted RNA pull-down assay to verify their physical association, in which biotinylated NC probe (biotin-NC) and miR-671-5p (biotin-miR-671-5p) were transfected into A549 and H1229 cells. In both cell lines, circ_0017109 showed significant enrichment by biotin-miR-671-5p when compared to biotin-NC probe (Fig. 3B). Using an antibody against argonaute 2 (AGO2), we then performed RNA immunoprecipitation assay (RIP) in A549 and H1299 cells, with IgG isotype as the control. We observed that both miR-671-5p and circ_0017109 were significantly enriched by anti-AGO2 antibody (Fig. 3C). Collectively, these results provided further evidence for the physical binding and association between circ_0017109 with miR-671-5p in NSCLC cells.

### Circ_0017109 promotes FZD4 expression via sponging miR-671-5p

**Fig. 4:**
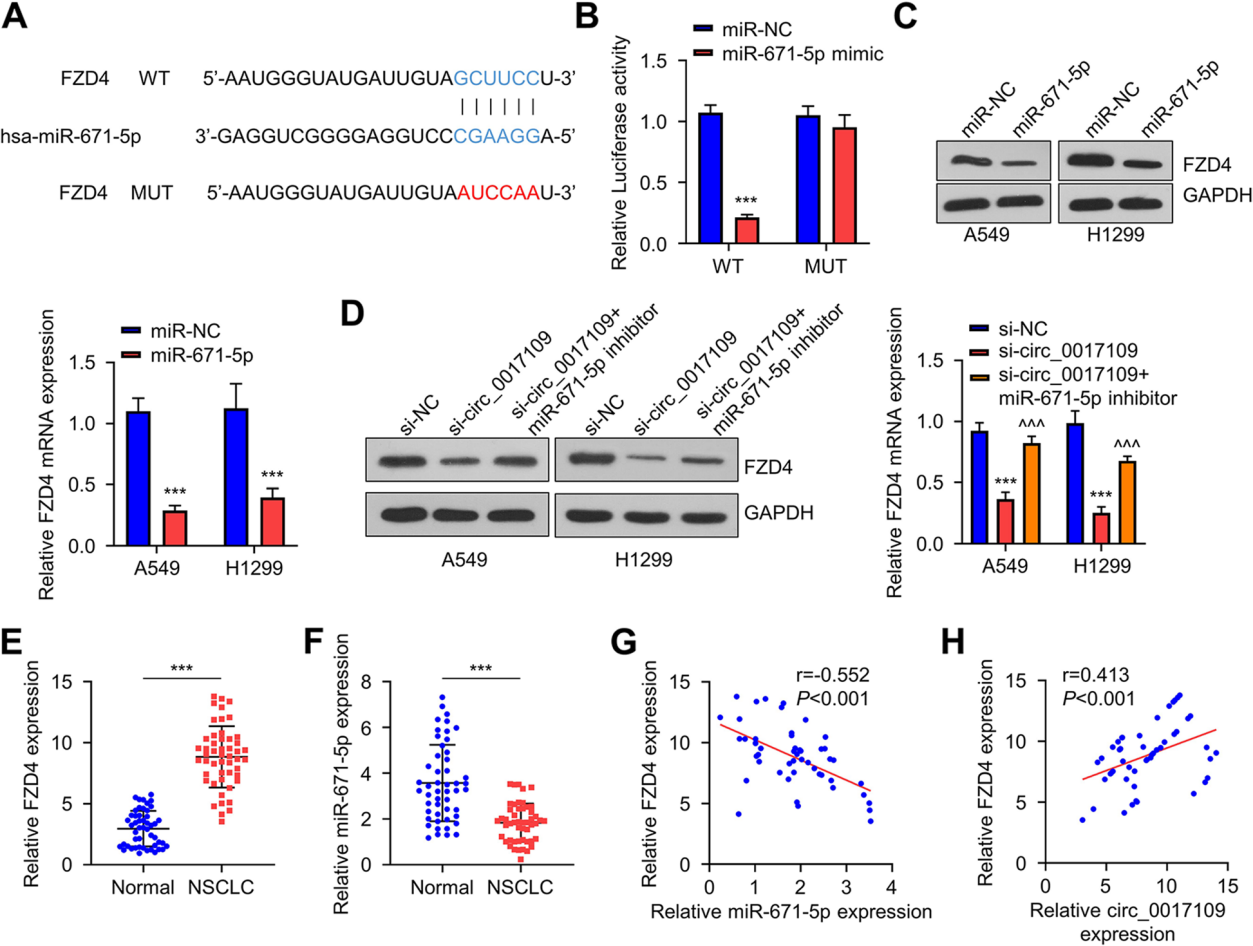
Circ_0017109 promotes FZD4 expression via sponging miR-671-5p. **A** miR-671-5p target sequence in the FZD4 3′-UTR was predicted using Targetscan database. **B** Luciferase activity of wild type or mutated FZD4 reporter in the presence of miR-NC or miR-671-5p mimic. **C** FZD4 mRNA and protein expression in H1229 and A549 cells after the transfection of miR-671-5p mimic. **D** The effect of circ_0017109 silencing and miR-671-5p inhibitor on FZD4 protein level. **E** Relative FZD4 expression in NSCLC tumor and normal lung tissue samples. **F** Relative miR-671-5p levels in NSCLC tumor and normal lung tissue samples. **G** Correlation of FZD4 expression and miR-671-5p in NSCLC tumor samples. **H** FZD4 level and circ_0017109 expression in NSCLC tumor samples. ***, *P* < 0.001 compared with si-NC group, and ^^^, *P* < 0.001 compared with si-circ_0017109 group

To search for the mRNA target of miR-671-5p, we used different online databases to predict the potential interacting mRNA of miR-671-5p. Through prediction, it is found that miR-671-5p could target FZD3, FZD4 and FZD7 in TargetMiner database (https://www.isical.ac.in/~bioinfo_miu/targetminer20.htm); miR-671-5p could potentially bind to FZD2 and FZD3 mRNA in Targetscan database (http://www.targetscan.org/vert_71/). We then performed qRT-PCR analysis of FZD2, FZD3, FZD4 and FZD7 mRNA in NSCLC cells and human bronchial epithelial HBE cells. The results showed that compared to control (HBE cells), FZD4 showed the highest expression in A549 and H1299 cells (Fig. S3). Since circ_0017109 upregulation in A549 and H1299 cells could sponge miR-671-5p, we therefore hypothesized that circ_0017109/ miR-671-5p axis regulates FZD4 expression. To investigate this hypothesis, we performed dual luciferase reporter assay using WT or MUT reporter of FZD4 binding sites as illustrated by Fig. 4A. The co-transfection of miR-671-5p mimic significantly decreased the luciferase activity of FZD4 WT reporter, but such inhibition was not detected using FZD4 MUT reporter (Fig. 4B). Accordingly, both mRNA and protein levels of FZD4 in H1229 and A549 cells showed downregulation upon the transfection of miR-671-5p mimic (Fig. 4C). Besides, circ_0017109 knockdown reduced the protein level of FZD4, while the co-transfection of miR-671-5p inhibitor partially rescued FZD4 level (Fig. 4D). To show their expression pattern in NSCLC tumors and normal tissues, we performed qRT-PCR to detect the expression of FZD4 and miR-671-5p. It was found that FZD4 level was increased while miR-671-5p level was reduced in NSCLC tumor samples (Fig. 4E and F). Additionally, relative FZD4 level was positively related to circ_0017109 level but negatively related to miR-671-5p expression (Fig. 4G and H). Altogether, these data suggest that the sponging activity of circ_0017109 on miR-671-5p consolidates FZD4 expression.

### Circ_0017109 promotes the proliferation and survival of NSCLC cells through modulating miR-671-5p/FZD4 axis

**Fig. 5:**
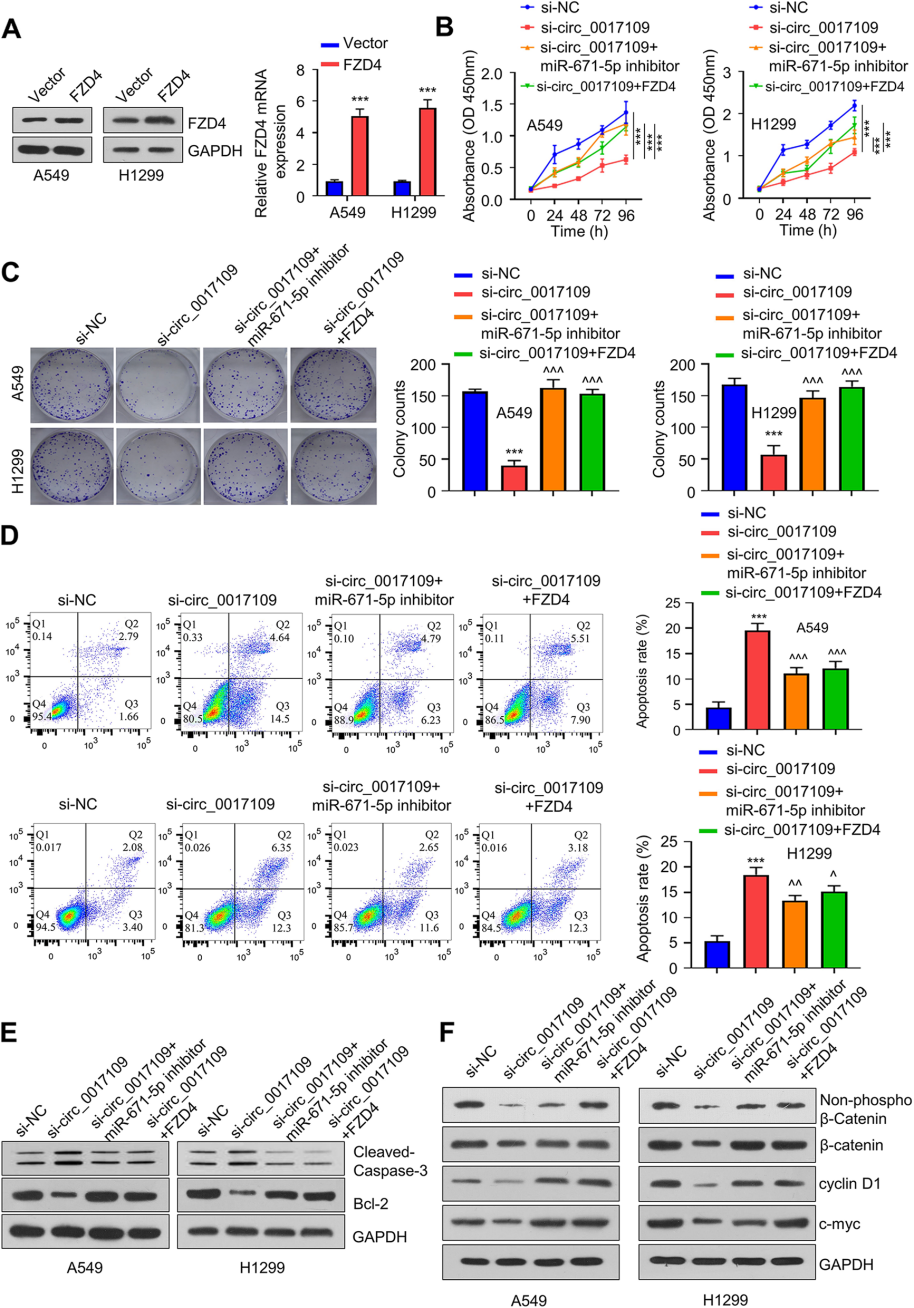
Circ_0017109 modulates miR-671-5p/FZD4 axis and Wnt/β-catenin pathway. **A** FZD4 overexpression in H1229 and A549 cells upon the transfection of FZD4 expression vector. **B** CCK-8 assay in A549 and H1299 cells transfected with si-circ_0017109, si-NC, si-circ_0017109 + miR-671-5p inhibitor, or si-circ_0017109 + FZD4. **C** Colony forming assays in A549 and H1299 cells transfected with si-circ_0017109, si-NC, si-circ_0017109 + miR-671-5p inhibitor, or si-circ_0017109 + FZD4. **D** Apoptosis in A549 and H1299 cells transfected with si-circ_0017109, si-NC, si-circ_0017109 + miR-671-5p inhibitor, or si-circ_0017109 + FZD4. **E** Cleaved-caspase-3 and Bcl-2 levels within H1229 and A549 cells post-transfection. **F** Protein levels of FZD4, Bcl-2, β-catenin, non-phospho β-catenin, c-myc, cyclin D1, and cleaved caspase-3 in A549 and H1299 cells transfected with si-circ_0017109, si-NC, si-circ_0017109 + miR-671-5p inhibitor, or si-circ_0017109 + FZD4. **, *P* < 0.01, ***, *P* < 0.001 compared with si-NC group, ^, *P* < 0.05, and ^^^, *P* < 0.001 compared with si-circ_0017109 group

To show the role of FZD4 as a downstream effector of circ_0017109, pcDNA3.1-FZD4 expression vector was transfected into H1229 and A549 cells. Compared to the empty vector (control), FZD4 vector significantly increased FZD4 mRNA and protein levels (Fig. 5A). Knockdown of circ_0017109 significantly reduced A549 and H1299 cell growth, and both miR-671-5p inhibitor and FZD4 overexpression restored cell growth capacity (Fig. 5B). We also observed a 70% reduction in clonogenic ability of A549 and H1299 cells after circ_0017109 knockdown, and both miR-671-5p inhibitor and FZD4 overexpression rescued the colony formation capacity (Fig. 5C). Consistently, apoptosis induction by circ_0017109 knockdown in A549 and H1229 cells were suppressed by miR-671-5p inhibitor and FZD overexpression (Fig. 5D), which was further evidenced by the changes in cleaved caspase-3 and Bcl-2 protein levels (Fig. 5E). As FZD is an upstream regulator of WNT/β-catenin signaling, we also examined the protein changes in this pathway. It was found that circ_0017109 silencing reduced the level of β-catenin, non-phospho (active) β-catenin, as well as the positive regulators of cell cycles including cyclin D1 and c-Myc. (Fig. 5F). The co-transfection of miR-671-5p inhibitor or FZD overexpression increased the expression levels of these proteins. Together, these finding imply that circ_0017109/ miR-671-5p axis modulates WNT/β-catenin signaling by targeting FZD4.

### Circ_0017109 promotes oncogenesis of NSCLC cells in mouse model

**Fig. 6:**
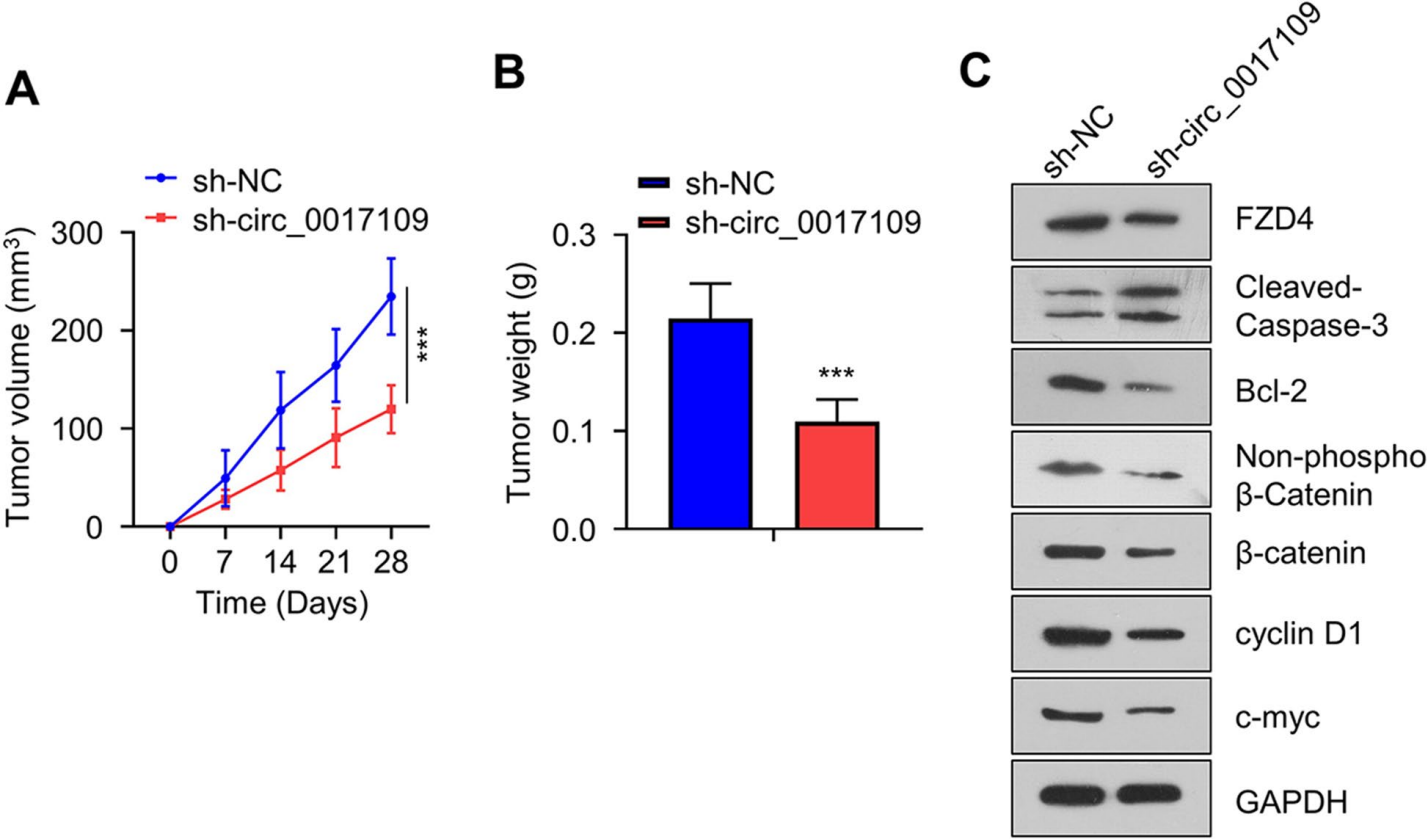
Xenograft tumorigenesis assay in nude mice. **A** Subcutaneous xenograft volumes and **B** weights were measured in mice injected with A549 cells stably expressing sh-NC and shRNA targeting circ_0017109. **C** Protein levels of cleaved caspase-3, FZD4, β-catenin, non-phospho β-catenin, Bcl-2, c-myc and cyclin D1 in xenograft tumor tissues with sh-NC or shRNA targeting circ_0017109. ***, *P* < 0.001

The xenograft mouse model of NSCLC cells were established in nude mice using A549 cells stably expressing sh-NC (scramble shRNA) or shRNA targeting circ_0017109. We found that A549 cells with stable silencing of circ_0017109 showed retarded tumor growth ability and resulted in less tumor tissues in nude mice (Fig. 6A and B). We also performed WB using xenograft tumor tissues to analyze WNT/FZD4/β-catenin singling process. We found that in tumor tissues with circ_0017109 knockdown, FZD4, β-catenin and non-phospho β-catenin, cyclin D1, and c-Myc showed significant reduction, and a higher level of cleaved caspase-3 and a lower level of Bcl-2 were also detected (Fig. 6C). These findings were consistent with in vitro cell experiments, indicating that downregulation of circ_0017109 inhibits the tumorigenesis of A549 cells by targeting FZD4 and WNT/β-catenin signaling.

## Discussion

The relevance of circRNAs in human cancer has drawn intensified research attention, and over 100,000 unique human circRNAs have been annotated [18, 19]. However, their functional characterization is far from completion in the context of cancer. The expression pattern and regulatory network of circRNAs in human cancer remain largely unexplored. The present study focused on the delineation of the functional role and regulatory mechanism of circ_0017109 in NSCLC. Circ_0017109 showed an overexpression within NSCLC cells and tissues, and there was a correlation between its high expression and poor overall survival in NSCLC cases. Notably, based on our in vivo and in vitro results, circ_0017109 was required to sustain the proliferation and survival of NSCLC cells by sponging miR-671-5p and maintaining the activity of FZD4/Wnt/β-catenin pathway.

A growing body of circRNAs were found to be aberrantly expressed in NSCLC, and they function as either oncogenes [20–26] or tumor suppressors [27–29]. For instance, amplification or overexpression of circRNAs, including circRNA_102231 [23], circPVT1 [20], ciRS-7/CDR1as [24], circRNA 100,146 [26], and circFGFR1 [25], can downregulate tumor suppressors or negative regulators in cell division, thereby contributing to uncontrolled proliferation and tumorigenesis in NSCLC. CircRNA-Foxo3, a downregulated circRNA in NSCLC, acts as a tumor-suppressor to negatively target oncogenic miR-155 [28]. CircRNAs such as circ_0001649 [27] and circPTPRA [29], are expressed at a relatively low level in NSCLC to produce anti-proliferative, anti-tumorigenic, and pro-apoptotic effects on NSCLC cells. Notably, the functional roles of circRNAs are quite diversified in different cancers. CircRNA-Foxo3, for example, has been shown to be expressed at different levels and exert discrepant roles in five different cancers (breast [30], esophageal squamous cell [31], prostate [32], bladder [33], and urothelial carcinoma [34]). The underlying mechanisms of their aberrant expression and functions are unclear at present. Previously, Tan et al. reported that a tumor-promoting circRNA (F-circEA-4a) was generated from an oncogenic fusion gene (EML4-ALK) in NSCLC [35], and the altered expression of this circRNA may arise from chromosomal translocations. Other potential mechanisms, such as alterations in *cis*-elements, transcription, and spliceosomal machinery have also been suggested for the deregulation of circRNAs [36]. It remains to be explored how circ_0017109 is upregulated in NSCLC cells.

To acts as the molecular sponge of miRNAs is the most frequently described role of circRNAs. This function was first identified in a seminal study by Hansen and colleagues, in which ciRS-7 adsorbs miR-7 and results in increased expression of miR-7 targets. In NSCLC, many of aberrantly expressed circRNAs have been shown to act as a miRNA sponge. For instance, the oncogenic circRNA_100,146 contains binding sites for miR-361-3p and miR-615-5p [26], whereas tumor suppressor circ_0001649 can absorb miR-331-3p and miR-338-5p [27]. Similarly, in our study, we showed that circ_0017109 serves as miR-671-5p sponge to negative regulate its activity. We further demonstrated circ_0017109/miR-671-5p regulates the expression of FZD4, thereby modulating the activity of Wnt/β-catenin signaling. CircRNA may also regulate epithelial-mesenchymal transition (EMT) in cancer cells. For instance, circPTK2 can inhibit the oncogenic activity of miR-429 and miR-200b-3p to suppress EMT and tumor metastasis [37]. Wnt signaling pathway is frequently deregulated in cancers, and contributes to EMT [38]. In NSCLC study, previous studies showed that the downregulation of miR-3127-5p regulates Wnt/FZD4/β-catenin signaling axis and promotes mesenchymal transformation [39], and it was reported that high FZD4 expression level is related to the poor prognosis of NSCLC [40]. Whether circ_0001649 also modulates EMT process and cell invasion in NSCLC cells warrants further investigation.

To conclude, we reported the overexpression of circ_0017109 in NSCLC and its upregulation may contribute to the development of NSCLC by promoting cell proliferation and inhibiting apoptosis. In NSCLC cells, circ_0017109 acts as the molecular sponge to downregulate miR-671-5p while activating FZD4/Wnt/β-catenin signaling. Collectively, these results uncover a novel role of miR-671-5p in the progression of NSCLC, indicating that circ_0017109 may be employed as the diagnostic and prognostic biomarker for NSCLC patients.

## Material and methods

### Human NSCLC tissues

A total number of 50 NSCLC tumor samples and matched non-carcinoma lung samples were collected at the Department of Lung Cancer, Tianjin Medical University Cancer Institute and Hospital (Tianjin, China). This study was approved by the medical research ethics committee of Tianjin Medical University Cancer Institute and Hospital. All the patients signed the informed consent before surgery. The NSCLC tumor samples and adjacent normal samples were collected and snap-frozen in liquid nitrogen freezing before the preservation under − 80 °C.

### Cell culture and transfection

Human bronchial epithelial cells (HBE) and human NSCLC cells (CALU3, CALU6, A549, H1229), and human renal epithelial cell line (H293T) were acquired from American Type Culture Collection (Virginia, USA). Cells were cultured win DMEM that contained 10% FBS (Gibco, NY, USA) and 100 U/ml of penicillin and 100 μg/ml of streptomycin (Sigma, Germany) in a humid incubator under 5% CO_2_ at 37 °C. pcDNA3.1-FZD4 expression plasmid, small interfering RNA (siRNA) of circ_0017109, control siRNA, miR-671-5p inhibitor, miR-671-5p mimics and the corresponding negative controls (NCs) were provided by Genomeditech Co., Ltd. (Shanghai, China). Transfections were performed with Lipofectamine 2000 (Invitrogen) in line with specific instructions. Briefly, in 6-well plates, 60% confluent cells were transfected with 6 μg of pcDNA3.1-FZD4, or 50 nM of miRNA mimic, inhibitor or siRNA according to manufacturer’s instruction. After transfection for 48-h, the cells were collected for further experimental analysis.

### Quantitative real-time PCR (qRT-PCR)

TRIzol reagent (Invitrogen, USA) was employed for RNA extraction. 1 μg of RNA sample was prepared into cDNA using PrimeScript II 1st Strand cDNA Synthesis Kit (TaKaRa, Tokyo, Japan) in line with specific protocols. CFX96 Touch Real-time PCR detection system (Bio-Rad, Hercules) was adopted for qPCR analysis using specific primers and SYBR Premix Ex Taq II (Takara). GAPDH or U6 was used as controls for protein-coding gene or non-coding RNA respectively, and the results were analyzed using the 2^−ΔΔCT^ method. For miR-671-5p, reverse transcription was performed using Taqman MiRNA Reverse Transcription Kit (Applied Biosystems, MIMAT00038800). The primer sequences used in the study were as follows: circ_0017109, 5′-AGATTCCGTTCGGCTCCTCC-3′ (F) and GCACTGGTGTCTGTTG TTAGC-3′ (R); FZD4, 5′-CCTCGGCTACAACGTGACC-3′ (F) and 5′-TGCACATT GGCACATAAACAGA-3′ (R); U6, 5′-GACAGTCAGCCGCATCTTCT-3′ (F) and 5′-GCGCCCAATACGACCAAATC-3′ (R); GAPDH; 5′-CTCGCTTCGGCAGCACA-3′ (F) and 5′-AACGCTTCACGAATTTGCGT-3′ (R).

### Treatment with RNase R

A total amount of 5 μg RNA was equally divided into two portions: one was treated with 3 units of RNase R (Epicenter, Madison, WI) under 37 °C for 15 min, and the other was incubated at the same condition without RNase R (Mock). After treatment, RNA samples were collected by RNeasy MinElute Cleaning Kit (Qiagen, Germany), which was further analyzed via qRT-PCR.

### Subcellular fractioning

Nuclear and Cytoplasmic RNA Purification Kit (Norgenbiotek Corporation, Thorold, Canada) was adopted for the nuclear and cytoplasmic fractioning, and the subsequent RNA isolation from A549 and H1299 cells. Circ_0017109 expression levels in different fractions were evaluated through qRT-PCR, with U6 and GAPDH being nuclear and cytoplasmic references, respectively.

### Colony formation and CCK-8 proliferation assay

For colony formation experiment, cells (1000/well) were seeded into the 6-cm culture dish, and cultured for 2 weeks with medium replenishment every 2 days. On day 14, cells were subjected to 4% paraformaldehyde (PFA) fixation and the staining of 0.4% crystal violet (Sigma, Germany) for 20 min. Colonies that contained over 10 cells were counted under light mode of an FV1000 microscope (Olympus, Tokyo, Japan), at 200X magnification.

CCK-8 kit (Abcam, ab228554) was utilized to detect cell proliferation following specific instructions. 48 h post transfection, 2000 cells were seeded into each well in 96-well plates and the cells were cultured for different durations. At indicated time point, the cells were mixed with CCK-8 reagent (20 μL/well), followed by 2-h incubation under 37 °C. The absorbance (OD) measurement was conducted using a microplate reader at 450 nm.

### Flow cytometry analysis of apoptosis

For quantifying apoptosis level, Annexin V-FITC/Propidium Iodide kit (BCT- Adipogen，XAP2102-TT01) was used to stain cells following the manufacturer’s instructions. Briefly, 1 × 10^6^ cells in 1 ml Annexin V staining buffer was mixed with 1 μl Annexin V dye and 1 μl Propidium Iodide reagent. After 15-min staining, the cells were washed twice with staining buffer and then analyzed using BD FACSCalibur™ Flow Cytometer (BD Biosciences).

### Western blotting (WB) assay

Protein samples collection was performed using RIPA buffer (KeyGen, Shanghia, China) on ice for 15 min. Total protein contents were quantified by an Enhanced BCA Protein Assay Kit (Beyotime, Beijing, China). Aliquots of protein were loaded onto 10% SDS-PAGE gel, followed by transfer onto PVDF membranes (Millipore, Darmstadt, Germany). The membranes were subjected to blocking using 5% bovine serum albumin (BSA) for 1 h. The incubation with primary antibodies was performed at 4 °C overnight: cleaved caspase-3 (1:2000, Abcam, ab2302), Bcl-2 (1:2000; Abcam, ab59348), FZD4 (1:1500; Bioss, bs-13217R), β-catenin (1:1500; Bioss, bs-1165R), non-phospho (Active) β-catenin (1:1000; Cell Signaling Technologies, 8814), cyclin D1 (1:2000; Abcam, ab134175), c-Myc (1:1500; Abcam, ab190026), or GAPDH (1:5000; Bioss, bs-2188R). The next day, the membranes were washed 4 times with TBST buffer and then further labeled with HRP-linked secondary antibody (1:10000; Invitrogen, 61–6520) for 1 h at room temperature. After rinsing for 4 times signal development was conducted using a chemiluminescence kit (Santa Cruz, TX, USA), and the protein bands were photographed under a gel imager (Biorad, CA, USA).

### Luciferase reporter assay

H293T cells were used for dual luciferase reporter assay. In 96-well plates, cells were transfected with dual luciferase reporter with wild type (WT binding site or the reporter with mutated binding sequences (MUT), in the presence of miR-671-5p mimics or miR-NC. After 48 h, Luciferase Reporter Assays Substrate Kit (Abcam, ab228546) was adopted for measuring the relative luciferase activities of Firefly and Renilla luciferase in line with specific protocols.

### RNA pull-down assay

Cells were transfected with 50 nM of biotinylated negative control (bio-NC) or miR-671-5p mimics (bio-miR-671-5p) using Lipofectamine RNAiMAX Transfection Reagent (Invitrogen, USA). 48 h post transfection. Cell lysate was collected using IP lysis buffer (Beyotime, Beijing, China), with 10% lysate being saved as the input sample. The remaining sample was mixed with 50 μl C-1 streptavidin magnetic beads (Invitrogen, USA) under 4 °C for 2 h. The beads were then washed 4 times with IP lysis buffer, followed by RNA sample purification with RNeasy Mini Kit (Qiagen, Germany). The relative level of circ_0017109 in each sample was determined by qRT-PCR.

### RNA immunoprecipitation

Cells were lysed using an Imprint® RNA Immunoprecipitation Kit in line with specific instructions (Sigma, Germany). One milliliter of cell lysate (1 million cells) was mixed with 5 μg anti-Ago2 or 5 μg IgG isotype, which was immobilized on protein-A magnetic beads. The mixture was incubated at 4 °C overnight. The beads were rinsed 3 times using the immunoprecipitation buffer, and the RNA samples associated with the beads were extracted using TRIzol reagent (Invitrogen, USA). qRT-PCR was employed for measuring the immunoprecipitated RNAs.

### Mouse xenograft assay

A549 cells with stable sh-circ_0017109 expression were generated by lentiviral transduction. pLKO.1-Puro lentiviral vector with circ_0017109 shRNA or scramble control shRNA (sh-NC) was provided by Genomeditech Co., Ltd. (Shanghai, China). The production of lentivirus was conducted in 293 T cells by GenePharma Co. Ltd. (Shanghai, China). Cells infected with lentivirus were selected with 800 ng/mL puromycin for 2 weeks before inoculation. Adult BALB/c nude mice (~ 25–30 g) were provided by National Resource Center for Mutant Mice of China (Nanjing, China). Nude mice were raised in a controlled environment at 22 °C with 12 h light/dark cycle. The animals were assigned to two groups (*n* = 6 each). To establish mouse xenograft model of NSCLC, each mouse was injected with 2 × 10^6^ A549 cells (stable expression of circ_0017109 shRNA or sh-NC) in PBS (150 μL) subcutaneously via flanks. Tumor size was measured every week using a caliber. After 4 weeks, euthanasia of mice was performed using 20% of carbon dioxide in a closed chamber for 15 min until no movement was observed. Tumor was then dissected for subsequent analysis. All the animal experimental protocols were conducted in compliance with animal use and care guidelines of Tianjin Medical University Cancer Institute and Hospital, and were approved by the corresponding animal experimental committee.

### Statistical analysis

SPSS19.0 and Prism (GraphPad Sofeware 7.0) were adopted for statistical analysis. Data were displayed as mean ± SD. Student’s t-test (two-tailed) was adopted for two group comparison, and one-way or two-way ANOVA was utilized for multiple comparisons. Pearson’s correlation coefficient was utilized to analyze associations of gene expressions. Kaplan Meier curve was plotted for assessing the overall survival of NSCLC patients, while results were examined using log-rank t-test. The different was considered as significant if *p* < 0.05.

## Supplementary Information

**Additional file 1: Fig. S1.** Circ_0017109 promotes cell growth and inhibits apoptosis of NSCLC cells. (A) Circ_0017109 expression within H1299 and A549 cells after the transfection of si-circ_0017109#2 or si-NC. (B) CCK-8 assay in H1299 and A549 cells after the transfection of si-circ_0017109#2 or si-NC. (C) Apoptosis detection in H1299 and A549 cells after the transfection of si-circ_0017109 or si-NC. **, *P* < 0.01, and ***, *P* < 0.001.

**Additional file 2: Fig. S2.** Validation of the expression of miRNA targets between NSCLC cells and HBE cells. (A) CircInteractome database (https://circinteractome.nia.nih.gov/) revealed multiple potential miRNA targets of circ_0017109. (B) qRT-PCR analysis of miRNA expression between NSCLC cells and HBE cells. All data were normalized to the expression level in HBE cells. ***, *P* < 0.001.

**Additional file 3: Fig. S3.** qRT-PCR analysis of FZD2, FZD3, FZD4 and FZD7 in NSCLC cells and HBE cells. All data were normalized to the expression level in HBE cells. *, *P* < 0.05, **, *P* < 0.01***, *P* < 0.001.

**Additional file 4.**

## Data Availability

The data-sets generated in this study are available from the corresponding author upon reasonable request.

## Abbreviations

BC: Breast cancer
CCK-8: Cell counting kit-8
circRNA: Circular ribonucleic acid
CRC: Colorectal cancer
FCM: Flow cytometry
FZD4: Frizzled-4
GBM: Glioblastoma
GC: Gastric cancer
K-M: Kaplan-Meier
LC: Lung cancer
LNM: Lymph node metastasis
NSCLC: Non–small cell lung cancer
qRT-PCR: Quantitative real-time PCR
WB: Western blotting
