# Supplementary figures and images for: CircRNA has_circ_0017109 promotes lung tumor progression via activation of Wnt/β-catenin signaling due to modulating miR-671-5p/FZD4 axis
Source: BMC Pulm Med. 2022 Nov 24;22:443. doi: 10.1186/s12890-022-02209-2 (PMC9700975; doi:10.1186/s12890-022-02209-2)

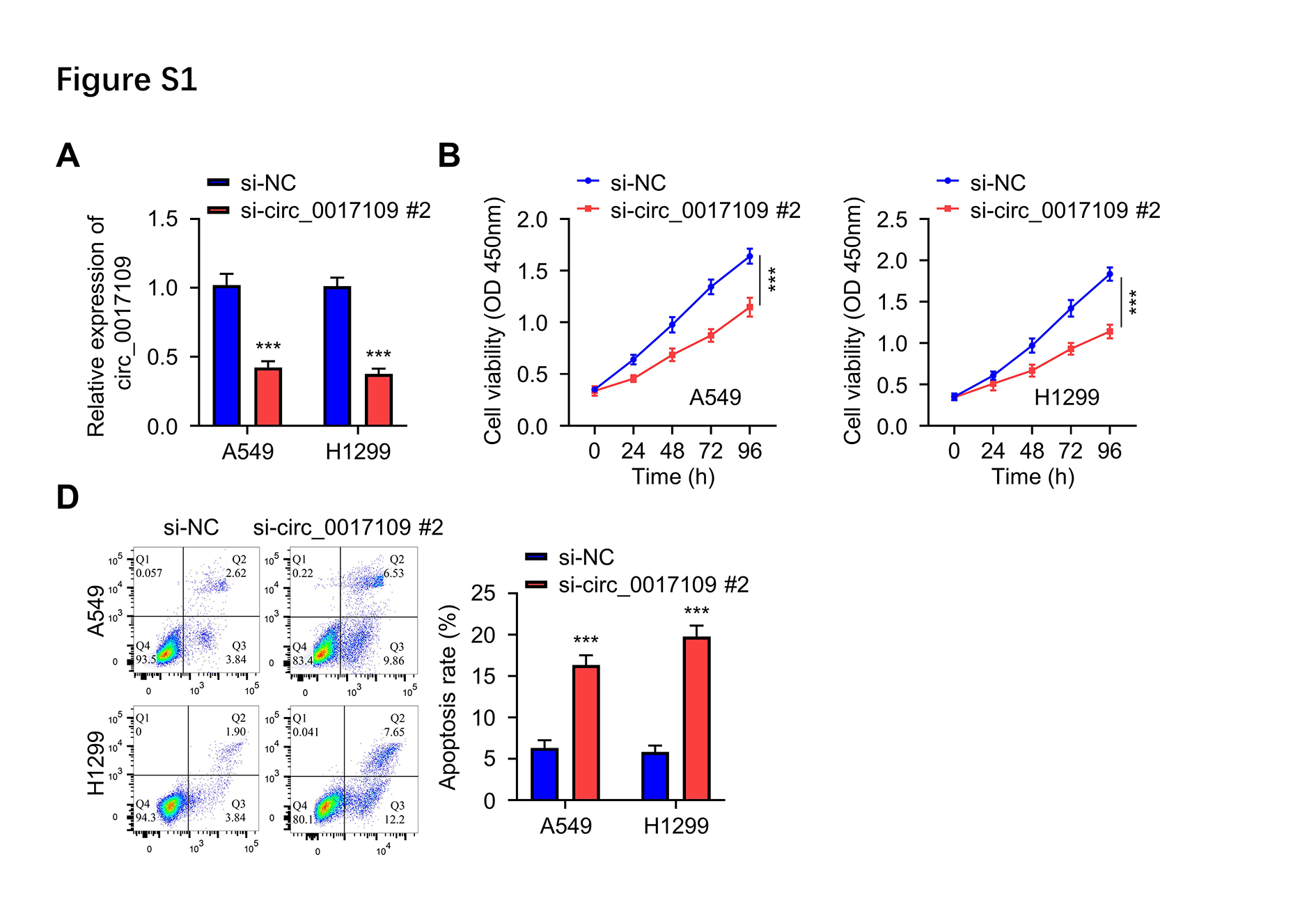

Supplement: Supplementary file 1 — Additional file 1: Fig. S1. Circ_0017109 promotes cell growth and inhibits apoptosis of NSCLC cells. (A) Circ_0017109 expression within H1299 and A549 cells after the transfection of si-circ_0017109#2 or si-NC. (B) CCK-8 assay in H1299 and A549 cells after the transfection of si-circ_0017109#2 or si-NC. (C) Apoptosis detection in H1299 and A549 cells after the transfection of si-circ_0017109 or si-NC. **, P < 0.01, and ***, P < 0.001. [file 12890_2022_2209_MOESM1_ESM.tif]

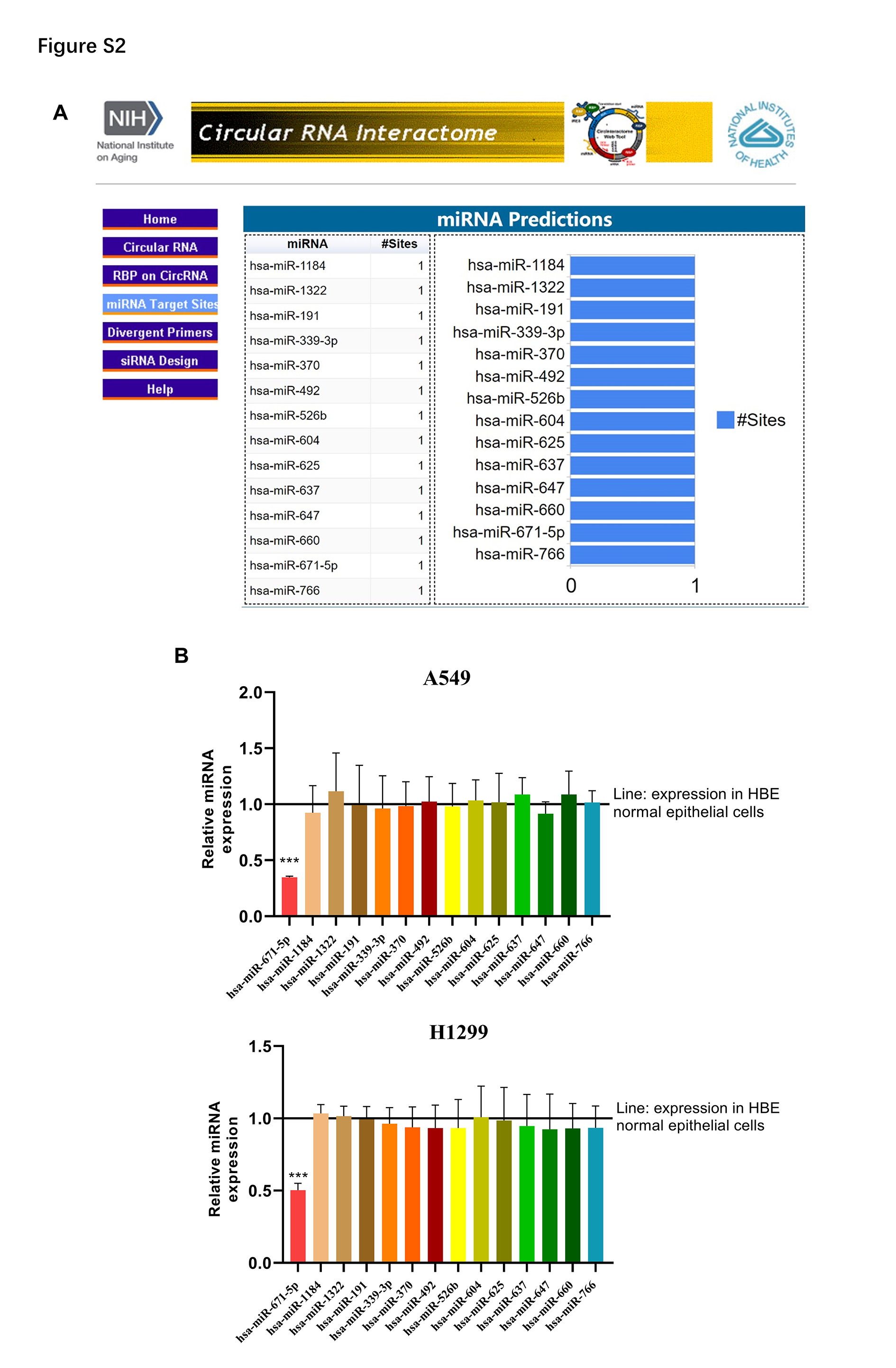

Supplement: Supplementary file 2 — Additional file 2: Fig. S2. Validation of the expression of miRNA targets between NSCLC cells and HBE cells. (A) CircInteractome database (https://circinteractome.nia.nih.gov/) revealed multiple potential miRNA targets of circ_0017109. (B) qRT-PCR analysis of miRNA expression between NSCLC cells and HBE cells. All data were normalized to the expression level in HBE cells. ***, P < 0.001. [file 12890_2022_2209_MOESM2_ESM.tif]

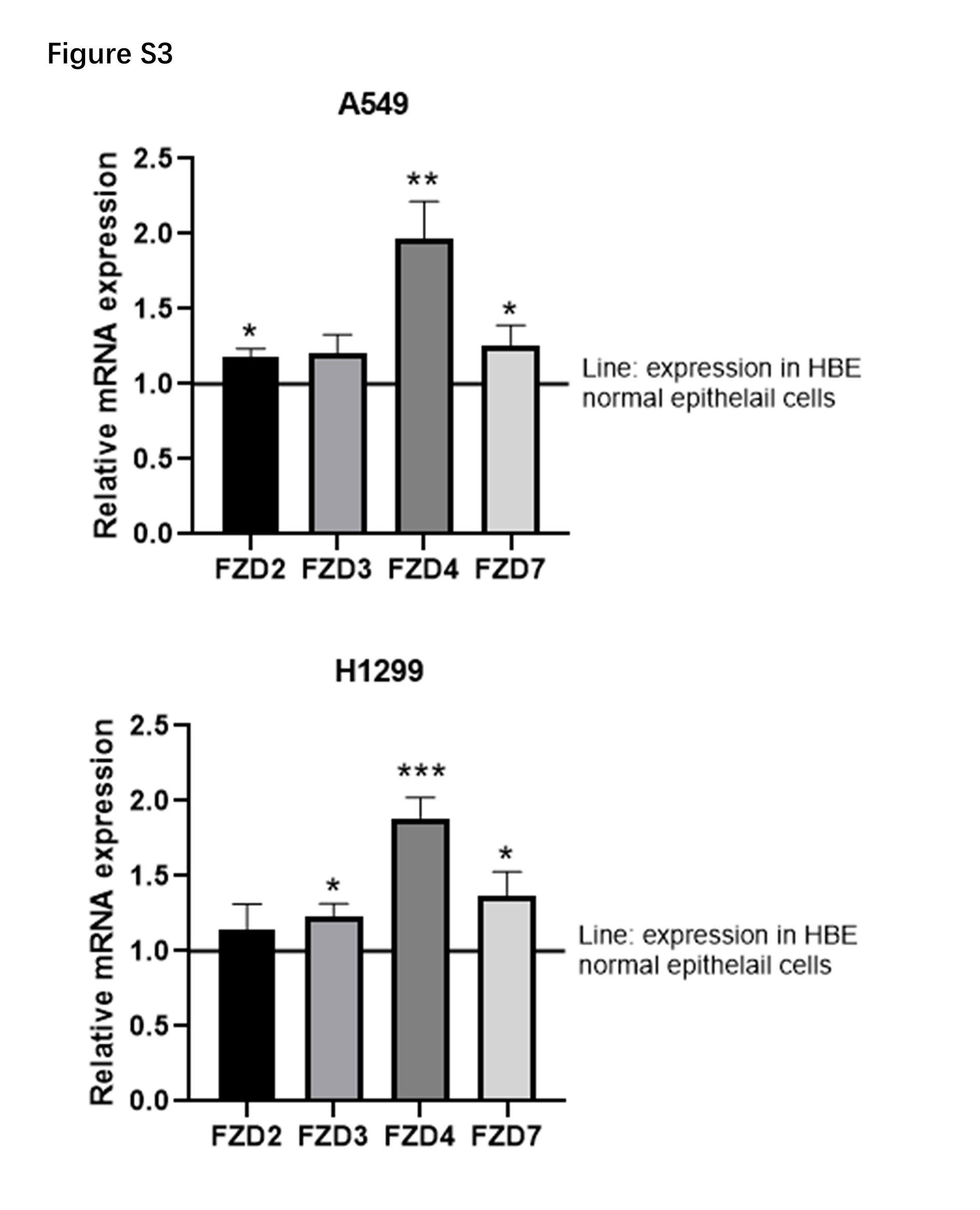

Supplement: Supplementary file 3 — Additional file 3: Fig. S3. qRT-PCR analysis of FZD2, FZD3, FZD4 and FZD7 in NSCLC cells and HBE cells. All data were normalized to the expression level in HBE cells. *, P < 0.05, **, P < 0.01***, P < 0.001. [file 12890_2022_2209_MOESM3_ESM.tif]
