# Supplementary material for: CircRNA has_circ_0017109 promotes lung tumor progression via activation of Wnt/β-catenin signaling due to modulating miR-671-5p/FZD4 axis
Source: BMC Pulm Med. 2022 Nov 24;22:443. doi: 10.1186/s12890-022-02209-2 (PMC9700975; doi:10.1186/s12890-022-02209-2)

Westernblot raw data for Fig 2E

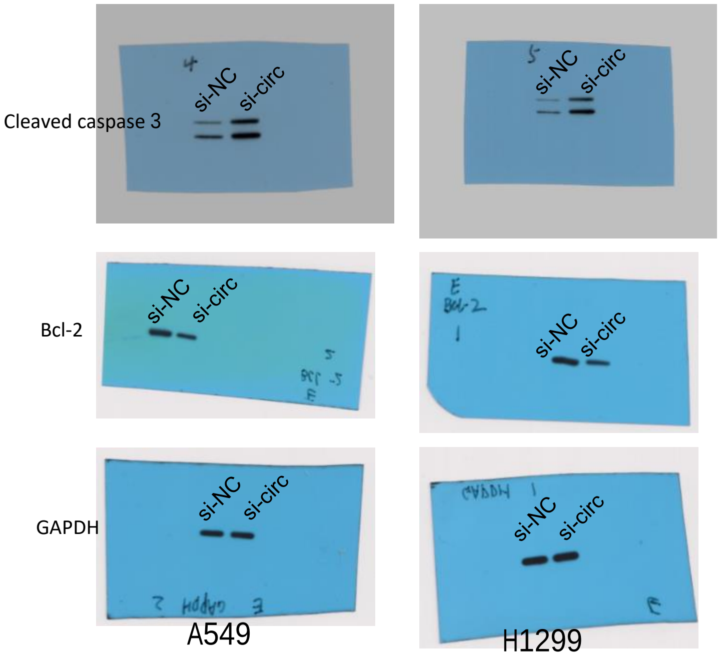

Westernblot raw data for Fig 5A

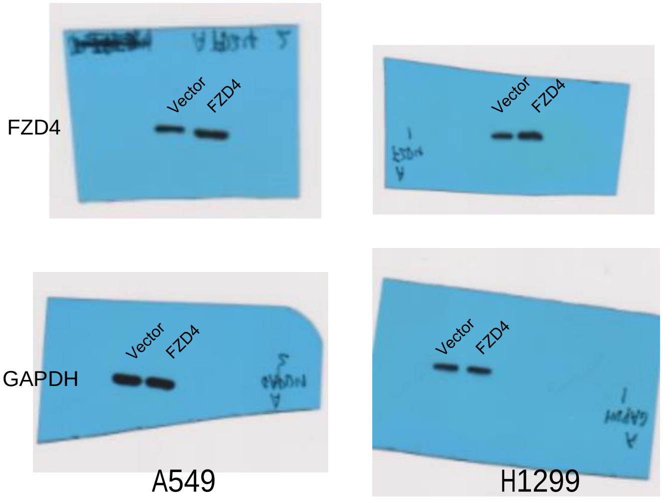

Westernblot raw data for Fig 4C

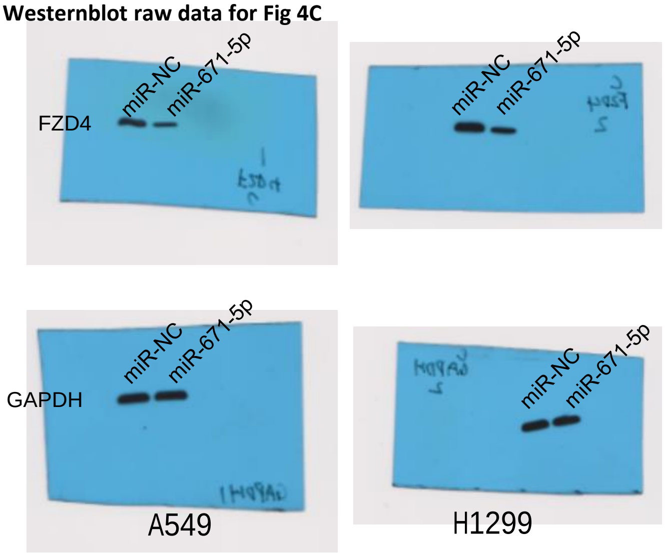

Westernblot raw data for Fig 4D

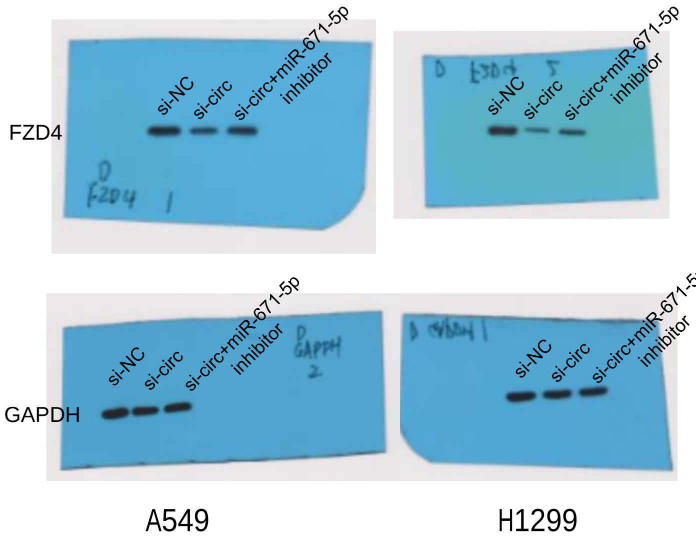

Westernblot raw data for Fig 5E

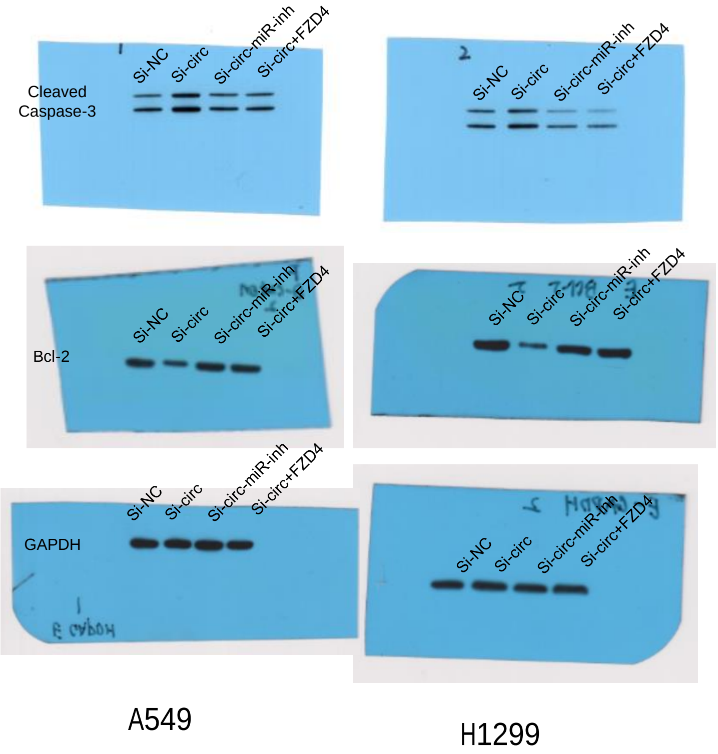

Westernblot raw data for Fig 5F

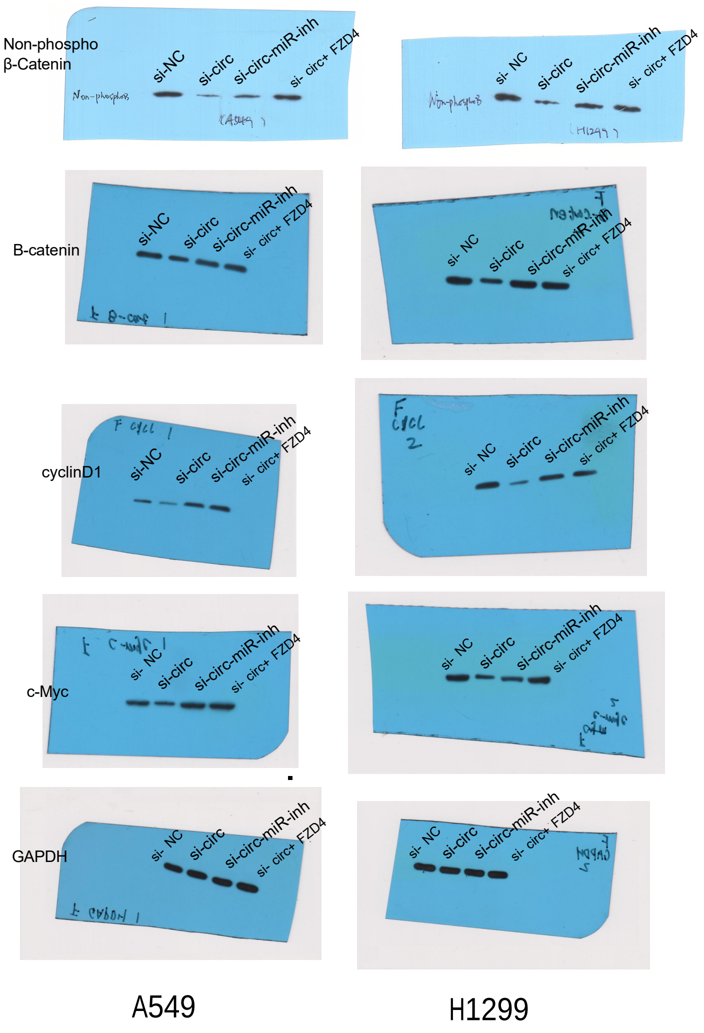

Westernblot raw data for Fig 6C

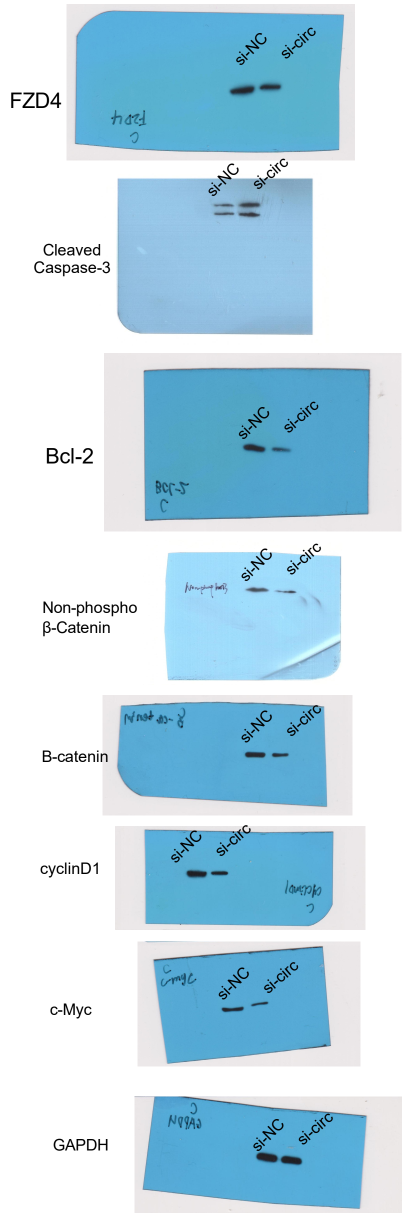

Supplement: Supplementary file 4 — Additional file 4. [file 12890_2022_2209_MOESM4_ESM.pdf]
